# Plasma Lipidomics Profiling Reveals Biomarkers for Papillary Thyroid Cancer Diagnosis

**DOI:** 10.3389/fcell.2021.682269

**Published:** 2021-06-21

**Authors:** Nan Jiang, Zhenya Zhang, Xianyang Chen, Guofen Zhang, Ying Wang, Lijie Pan, Chengping Yan, Guoshan Yang, Li Zhao, Jiarui Han, Teng Xue

**Affiliations:** ^1^Department of General Surgery, First Hospital of Tsinghua University, Beijing, China; ^2^BaoFeng Key Laboratory of Genetics and Metabolism, Beijing, China; ^3^Department of Oncology, Tai’an City Central Hospital, Tai’an, China; ^4^Zhongguancun Biological and Medical Big Data Center, Beijing, China

**Keywords:** papillary thyroid carcinoma, pathway, lipidomics, plasma samples, orthogonal partial least square discriminant analysis

## Abstract

The objective of this study was to identify potential biomarkers and possible metabolic pathways of malignant and benign thyroid nodules through lipidomics study. A total of 47 papillary thyroid carcinomas (PTC) and 33 control check (CK) were enrolled. Plasma samples were collected for UPLC-Q-TOF MS system detection, and then OPLS-DA model was used to identify differential metabolites. Based on classical statistical methods and machine learning, potential biomarkers were characterized and related metabolic pathways were identified. According to the metabolic spectrum, 13 metabolites were identified between PTC group and CK group, and a total of five metabolites were obtained after further screening. Its metabolic pathways were involved in glycerophospholipid metabolism, linoleic acid metabolism, alpha-linolenic acid metabolism, glycosylphosphatidylinositol (GPI)—anchor biosynthesis, Phosphatidylinositol signaling system and the metabolism of arachidonic acid metabolism. The metabolomics method based on PROTON nuclear magnetic resonance (NMR) had great potential for distinguishing normal subjects from PTC. GlcCer(d14:1/24:1), PE-NME (18:1/18:1), SM(d16:1/24:1), SM(d18:1/15:0), and SM(d18:1/16:1) can be used as potential serum markers for the diagnosis of PTC.

## Introduction

Thyroid cancer is the most common endocrine-related malignancy and the most prevalent cancer of the head and neck in the past decades (Omur and Baran, 2014). It accounts for 95% of all endocrine malignancies and 2.9% of all malignant diseases. The incidence of thyroid cancer has been ranked among the top 10 malignant neoplasms, including fifth place among female malignant neoplasms. It is estimated that 52,890 new cases of thyroid cancer are diagnosed in the United States each year (Siegel et al., 2020). Papillary thyroid carcinoma (PTC) is the most frequently common subtype of thyroid cancer (Hirsch et al., 2017), and discrimination of different types of thyroid cancers and benign nodules is currently carried out using various methods, usually in combination, namely, ultrasound, computed tomography, magnetic resonance imaging, cytology, fine needle aspiration (FNA), and surgery. FNA, being the most current effective preoperative method, still has its own challenges (Miccoli et al., 2012; Feldkamp et al., 2016). For example, it can cause harm to patients. The majority of PTC is indolent, but 1/3 of the patients still have persistent enlargement or recurrence and metastasis, so the benefit of distinguishing PTC patients is to closely follow up and monitor the PTC patients, so that the 1/3 patients can receive timely treatment (Bhargav et al., 2010).

At the same time, researchers have been searching for molecular markers that are valuable in diagnosing thyroid cancer, such as BRAF, RET/PTC, RAS, PAX8/PPARδ, P53, NTRK1, galectin-3. CK19, VEGF, Aurora-A, P16, AR, HBME-1, etc. (Grogan et al., 2010), but disappointingly, all these biomarkers either lack specificity to some extent or have a limited positive predictive value (Guo et al., 2015). Attempts are therefore still ongoing to identify a specific reliable biomarker. Moreover, a non-invasive screening method of thyroid malignancy remains unavailable.

Lipids played critical roles in cellular structures and functions, including cellular barriers, membrane matrices, signaling and energy storage. They undergo constant changes in physiological, pathological, and environmental conditions. Lipids play essential roles in cell growth and metabolism, therefore they are associated with carcinogenic pathways. Lipidomics, the metabolism of lipids, is defined as “the full characterization of lipid molecular species and of their biological roles with respect to expression of proteins involved in lipid metabolism and function, including gene regulation” (Zhao et al., 2015). First introduced by Han and Gross in 2003 (Han and Gross, 2003), lipidomics is an emerging system-based methodology for the systematic study of multiple lipids, and it helps to advance current knowledge in the field of lipid biology and steady-state. Lipidomics, by identifying alterations in cellular lipid metabolism, trafficking, and steady state, has been instrumental in determining the biochemical mechanisms of lipid-related disease. In recent years, it has been observed that many lipid species are significantly altered in patients with thyroid cancer (Ishikawa et al., 2012; Farrokhi Yekta et al., 2017), so that the lipid profile of the alterations may play a central role in the pathogenesis of thyroid carcinoma.

Recent advances in mass spectrometry (MS), nuclear magnetic resonance (NMR) and other spectroscopic methods have greatly facilitated the development and application of lipidomics (Hu et al., 2009), and MS has been used successfully either directly or in combination with chromatographic methods including ultra performance liquid chromatography-MS (UPLC-MS), gas chromatography-MS (GC-MS), and capillary electrophoresis-MS (CE-MS) to identify and quantify specific lipid species. In this study, we developed a UPLC-quadrupole time-polarization MS^*E*^ (UPLC-QTOF-MS^*E*^)-based technique for determination of total lipids present in patient plasma to identify the potential diagnostic biomarkers for thyroid cancer. UPLC-Q-TOF-MS has been used in systems analysis of complicated metabolome (Noh et al., 2016). Differential lipid metabolites between thyroid cancer patients and controls were identified by univariate and multivariate analysis. The identified biomarkers were validated and their diagnostic performance was accessed.

## Materials and Methods

### Patients and Study Design

Serum samples from PTC (*n* = 47) and control check (CK) (*n* = 33) were collected from the First Hospital of Tsinghua University from August 2016 to September 2019. The patients were selected according to the following criteria: (1) all patients with papillary thyroid carcinoma were diagnosed by pathology; (2) no patients received preoperative treatment, including adjuvant chemotherapy and radiotherapy; and (3) patients do not have hyperlipidemia, diabetes, and other diseases that might affect lipid metabolism. (4) Patients with a history of other malignancies or recurrent tumors were excluded. The selected healthy controls include age and gender-matched healthy subjects with no metabolic diseases and were proven to lack any lesions in thyroid after the physical examination followed by ultrasonography of the thyroid.

### Plasma Metabolite Extraction

Fasting venous blood samples were collected in EDTA anticoagulant tube. The fresh blood samples were transported to the laboratory for 20 min by cold chain (4°C), and the plasma was obtained by centrifugation at 1,000 g and 4°C. The plasma was cold extracted in a liquid nitrogen tank for 15 min, and then put into the –80°C freezer for analysis.

### Untargeted Metabolomic Detection

Mass spectrometry was an analytical method which ionizes the substance to be measured, separated it according to the mass/charge ratio of ions, and measured the intensity of various ion spectrum peaks to achieve the purpose of analysis. Mass was one of the inherent characteristics of substances. Different substances had different MS. Use this property, qualitative analysis (including molecular mass and related structural information) can be carried out. The peak intensity was also related to the content of the compound it represented and can be used for quantitative analysis.

### Data Processing and Statistical Analysis

Statistical analysis was conducted on clinical data, gender variables were analyzed using the chi-square test, and independent *t*-test was used for age variables. Metabolic changes in Plasma extract were analyzed by using UPLC-Q-TOF MS system and its software Progenesis QI (Waters). The original tandem mass spectrometry datasets were generated on the Waters XEVO-G2XS QTOF instrument and processed by the commercial software Progenesis QI 2.0, including raw data import, selection of possible adducts, peak set alignment, peak detection, deconvolution, dataset filtering, noise reduction, compound identification, and normalization with some method. The original data was preprocessed and the linear model was adjusted. Orthogonal Partial least squares discriminant analysis (OPLS-DA) was first used for classification discrimination. OPLS-DA was a supervised statistical method for discriminant analysis. OPLS-DA was used to establish a model of the relationship between the metabolite expression and the sample type, so as to realize the prediction of sample type (Worley and Powers, 2013). The reliability of the model was verified by cross validation and displacement test. The parameters R2 and Q2 were used to evaluate the interpretability and predictability of the model, respectively. By *P*-value (*P* < 0.05), VIP value (VIP > 1), and FDR value (FDR < 0.05) Standard potential difference marker is selected. The best truncation value was determined by using The Youden index. Finally, potential biomarkers were correlated with metabolic pathways through KEGG.

All statistical analyses were performed used R version 3.6.3, and *P* < 0.05 was considered statistically significant.

## Results

### Clinical Characteristics of the Subjects

There were 47 PTC patients (11 men and 36 women; age range, 23–72 years), and 33 healthy controls (6 women and 27 men; age range, 27 and 63 years). The clinical information of the samples was shown in Table 1.

**TABLE 1:** Baseline characteristics of the participants.

**Characteristic**	**CK**	**PTC**
	**(*n* = 33)**	**(*n* = 47)**
**Age, year**		
Mean ± SD	45.9 ± 10.1	45.5 ± 11.5
Range	27–63	23–72
**Sex, no. (%)**		
Female	27 (81.8)	36 (76.6)
Male	6 (18.2)	11 (23.4)
**Lymph nodes metastasis**		
Negative		21
Positive		26
**Stages**		
I		31
II		3
III		13
IV		0

**PTC*, papillary thyroid carcinoma; *CK*, healthy controls.*

### Plasma Metabolomics Profiles in the PTC and CK

#### Screening of Differential Metabolites in Plasma Samples Between the Two Groups

**FIGURE 1:**
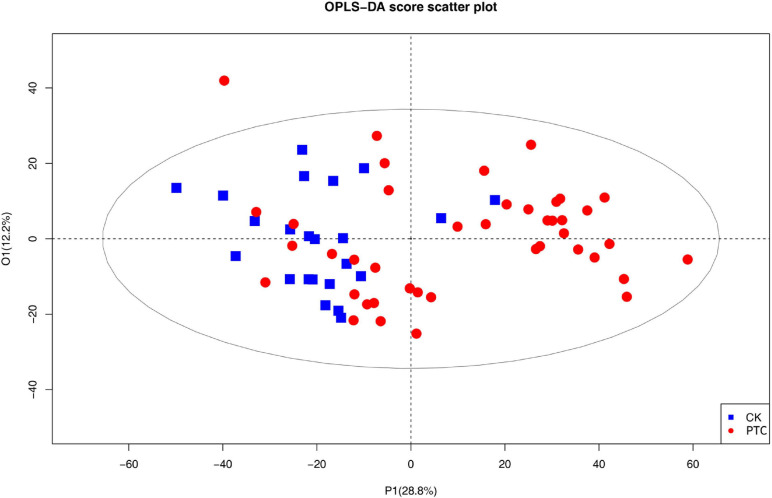
The OPLS-DA score plots based on Q-TOF data sets of metabolites in the plasma of patients with papillary thyroid carcinoma patient and healthy control groups. PTC represents one papillary thyroid carcinoma patient; CK represents one healthy control.

The data were originally divided into a *validation set* and a *training set.* To describe the changes between PTC group and CK group, an OPLS-DA model was developed (Figure 1).

**FIGURE 2:**
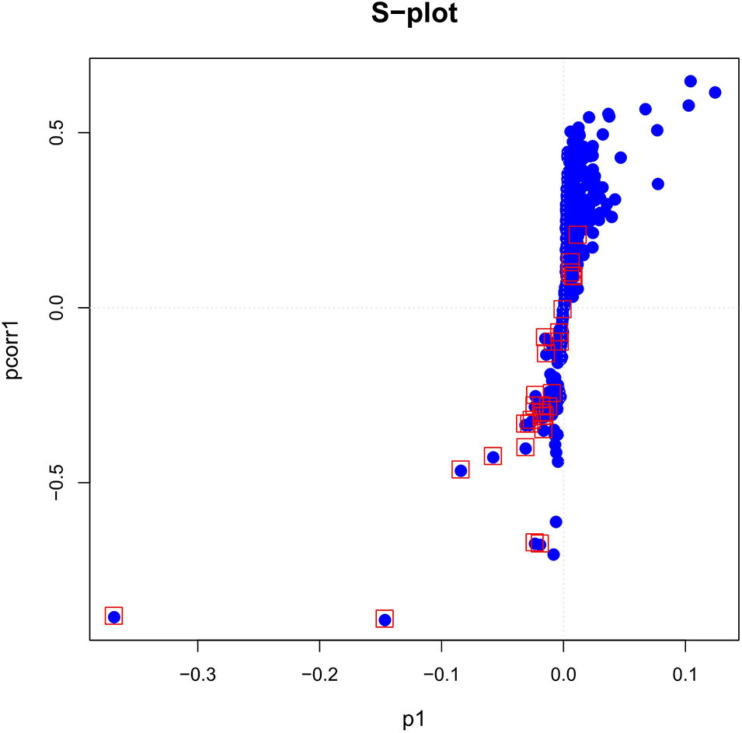
The S-plot based on *p*-values of correlation and covariance between metabolites in the plasma of patients with papillary thyroid carcinoma patient and healthy control groups.

As can be seen in the figure, the plasma lipid profile of the two groups changed significantly. In addition, we obtained the S-plot showing a good curve, and the further away the metabolites from the origin in the figure, the greater the contribution to the grouping (Figure 2). Thirty metabolites with VIP >1 were selected based on the variable importance projection (VIP) values in the OPLS-DA model. Univariate statistical analysis was performed using R project to further verify the statistical significance of the metabolite differences between the thyroid cancer group and the healthy control group (*P* < 0.05).

**FIGURE 3:**
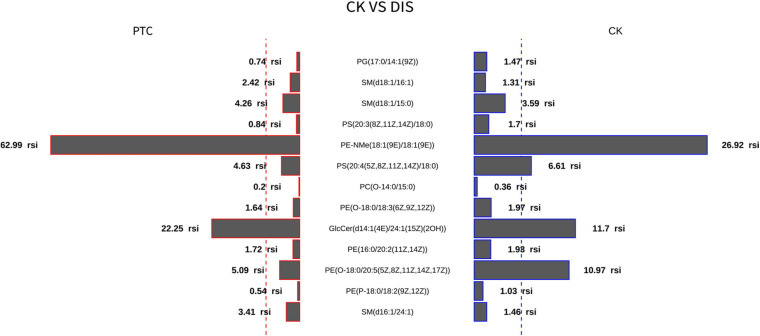
Butterfly diagram analysis of 13 different metabolites.

Thirteen metabolites with adjusted *P* < 0.05 were selected by the classic one-stage method (Table 2). The thirteen metabolites are PG(17:0/14:1), PE(16:0/20:2), PE(P-18:0/18:2), PE(O-18:0/20:5), SM(d18:1/15:0), PE(O-18:0/18:3), SM(d18:1/16:1), PS(20:3/18:0), GlcCer(d14:1/24:1), PC(O-14:0/15:0), SM(d16:1/24:1), PE-NMe(18:1/18:1), and PS(20:4/18:0). Butterfly diagram analysis (Figure 3) showed how these 13 lipid metabolites differed between thyroid cancer patients and healthy control populations. As can be seen from Figure 3, PTC group was significantly higher than CK group in SM(d18:1/16:1), SM(d18:1/15:0), PE-Nme (18:1/18:1), GlcCer(d14:1/1/24:1), SM(d16:1/24:1), and SM(d16:1/24:1), while CK group was significantly higher than PTC group in PG(17:0/14:1), PS(20:3/18:0), PS(20:4/18:0), and PE(O-18:0/20:5).

**TABLE 2:** Identified differentiating lipids between thyroid papillary cancer patients and healthy controls.

**No**	**Compounds**	**m/z**	**Class**	**VIP^*a*^**	**FC^*b*^**	***FDR*^*c*^**	***p*-value^*d*^**
1	PG(17:0/14:1(9Z))	707.49	Glycerophospholipids	13.15	0.07	0.007	0.000
2	PE(16:0/20:2(11Z,14Z))	742.54	Glycerophospholipids	3.51	0.02	0.036	0.036
3	PE(P-18:0/18:2(9Z,12Z))	728.56	Glycerophospholipids	2.05	4955.03	0.013	0.002
4	PE(O-18:0/20:5(5Z,8Z,11Z,14Z,17Z))	752.56	Glycerophospholipids	1.95	16.39	0.014	0.002
5	SM(d18:1/15:0)	687.54	Sphingolipids	1.79	0.28	0.062	0.023
6	PE(O-18:0/18:3(6Z,9Z,12Z))	748.52	Glycerophospholipids	1.65	0.13	0.066	0.026
7	SM(d18:1/16:1)	745.55	Sphingolipids	1.62	1.41E-08	0.082	0.036
8	PS(20:3(8Z,11Z,14Z)/18:0)	834.52	Glycerophospholipids	1.43	0.1	0.005	0.000
9	GlcCer(d14:1(4E)/24:1(15Z))	804.57	Sphingolipids	1.43	5E-04	0.013	0.002
10	PC(O-14:0/15:0)	700.53	Glycerophospholipids	1.37	2.86E + 08	0.013	0.002
11	SM(d16:1/24:1)	829.64	Sphingolipids	1.36	0.11	0.050	0.016
12	PE-NMe(18:1(9E)/18:1(9E))	802.56	Glycerophospholipids	1.32	0.37	0.039	0.011
13	PS(20:4(5Z,8Z,11Z,14Z)/18:0)	810.53	Glycerophospholipids	1.12	0.32	0.065	0.025

*^*a*^VIP value was obtained from OPLS-DA with a threshold of 1.0. ^*b*^FC value was calculated by the average value of the thyroid papillary cancer group divided by the average value of the healthy control group. FC with a value larger than 1 indicates a higher level of the compound in plasma of patients with thyroid papillary cancer, while a FC value lower than 1 indicates a lower level, compared to healthy controls. ^*c*^FDR, false discovery rate. ^*d*^*p*-values are calculated from the Wilcoxon rank-sum test.*

#### Using the Youden Index Formula to Select the Best Cut-off Values

To further assess the diagnostic performance of the lipid species identified, we selected the Youden analysis. As shown in Table 3, variables with the Youden index greater than 0.6 are selected for model analysis, including GlcCer(d14:1/24:1), PE-NMe(18:1/18:1), SM(d16:1/24:1), SM(d18:1/15:0), and SM(d18:1/16:1).

**TABLE 3:** Diagnostic performance of serum biomarkers in discriminating papillary thyroid carcinoma from healthy controls.

**No**	**Metabolites**	**Youden**
1	GlcCer(d14:1/24:1)	0.707
2	PE-NMe(18:1/18:1)	0.669
3	SM(d16:1/24:1)	0.659
4	SM(d18:1/15:0)	0.651
5	SM(d18:1/16:1)	0.639
6	PE(16:0/20:2)	0.362
7	PE(O-18:0/18:3)	0.353
8	PS(20:4/18:0)	0.352
9	PE(O-18:0/20:5)	0.299
10	PC(O-14:0/15:0)	0.292
11	PE(P-18:0/18:2)	0.291
12	PG(17:0/14:1)	0.266
13	PS(20:3/18:0)	0.248

#### Development and Validation of a Predictive Model

**FIGURE 4:**
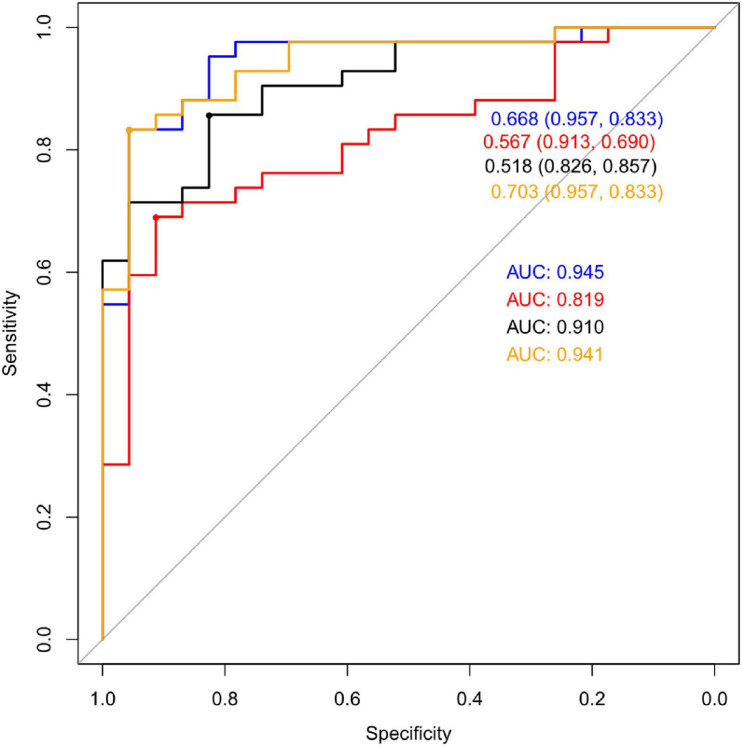
ROC curves: purple: LG; red: the SVM; black: RF; yellow: GBM.

Multivariate statistical analysis was used for further study. We chose Logistic Regression (LG), Recursive Partitioning (RPART), Support Vector Machine (SVM), Random Forest (RF), Gradient Boosting Machine (GBM) as the alternative algorithm. Through the 7-fold cross-validation, the indexes of each model were calculated, including accuracy, sensitivity, specificity and AUC. Statistical analysis of the results of 7-fold cross- validation showed that the classification effect of Logistic Regression was similar to that of SVM, which showed high AUC valued and high accuracy (Table 4). Validation set of the aforementioned model was shown in Figure 4. It can be seen from the figure that the AUC value of LG model was the highest: 0.945.

**TABLE 4:** Calculation of accuracy, sensitivity, specificity, and AUC after 7-fold cross-validation for different classifiers.

	**Accuracy**	**Sensitivity**	**Specificity**	**AUC**
LG	73.81	0.727	0.739	0.811
DT	59.84	0.391	0.713	0.679
SVM	64.45	0.56	0.69	0.713
RF	69.37	0.536	0.784	0.757
GBM	58.41	0.391	0.689	0.589

**LG*, logistic regression; *DT*, decision tree; *SVM*, support vector machine; *RF*, random forest; *GBM*, gradient boosting machine.*

### Pathway Analysis

**FIGURE 5:**
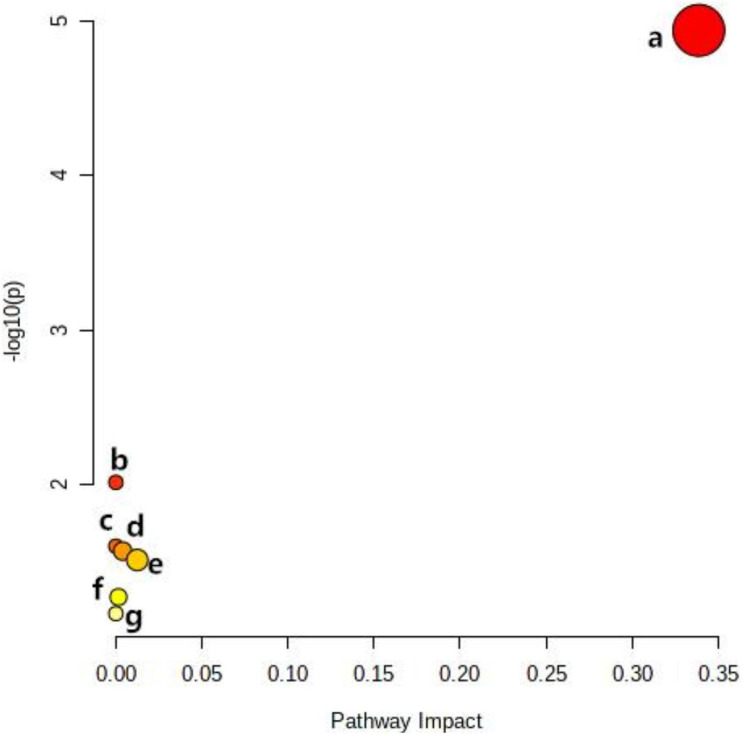
Ingenuity pathway analysis based on the 13 lipid metabolites with higher diagnostic performance. The *p*-value (y-axis) was represented by the color of the circle and the pathway impact (x-axis) was indicated by the size of the circle. Glycerophospholipid metabolism (a), Linoleic acid (b), alpha-Linolenic acid metabolism (c), Glycosylphosphatidylinositol (GPI) (d), Glycerolipid metabolism (e), Phosphatidylinositol signaling system (f), and Arachidonic acid metabolism (g).

**FIGURE 6:**
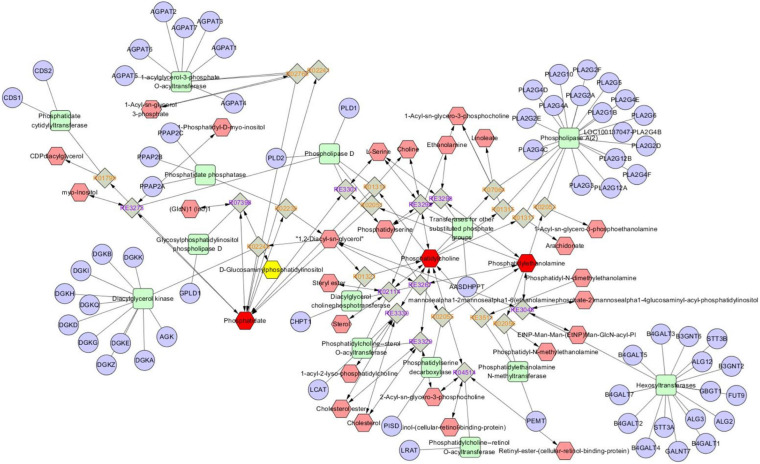
Network of the remarkably perturbed metabolic pathways in IBD by MetScape analysis. The red hexagons indicate the differential lipid metabolites identified in our study. And the pink ones are the involved metabolites not been identified in our study. The significant changed metabolites (*p* < 0.05) in IBD were shown as green line hexagons. The fold change of metabolites was indicated by hexagon’s size.

Metabolomics Pathway Analysis (MetPA) is a part of many functions of MetaboAnalyst network database. It can visualize the metabolic pathway information of potential biomarkers with the help of METLIN, HMDB, and KEGG database. As shown in Figure 5, the top seven dysregulated lipid pathways in thyroid cancer, as assessed by *p*-value or pathway impact, were associated with Glycerophospholipid metabolism (a), Linoleic acid (b), alpha-Linolenic acid metabolism (c), Glycosylphosphatidylinositol (GPI) (d), Glycerolipid metabolism (e), Phosphatidylinositol signaling system (f), and Arachidonic acid metabolism (g). Table 5 shows the details of each pathway. There were three different metabolites involved in glycerol metabolism pathway. Also, the network of significantly perturbed metabolomic data associated with differential lipids is summarized in Figure 6.

**TABLE 5:** The main metabolic pathways of biomarkers.

**Pathway name**	**Hits/Total**	***p*-value**	**FDR**	**–log(p)**	**Impact**
Glycerophospholipid metabolism	3/36	1.15E-05	0.000968	4.9383	0.33882
Linoleic acid metabolism	1/5	0.009652	0.4054	2.0154	0
alpha-Linolenic acid metabolism	1/13	0.024967	0.51524	1.6026	0
Glycosylphosphatidylinositol (GPI)	1/14	0.02687	0.51524	1.5707	0.00399
Glycerolipid metabolism	1/16	0.030669	0.51524	1.5133	0.01246
Phosphatidylinositol signaling system	1/28	0.053254	0.74556	1.2736	0.00152
Arachidonic acid metabolism	1/36	0.068115	0.81737	1.1668	0

*Total: the number of all metabolites in the metabolic pathway. Hits: the number of differentiated metabolites selected in the metabolic pathway. *p*-value: the original calculated *P*-value of the enrichment analysis. FDR: the value of FDR in multiplex checking. Impact: the influence value calculated by path topology analysis.*

## Discussion

In our study, UPLC-Q-TOF MS metabolomics technology was used to analyze the plasma of PTC group and CK group. Based on the classical statistical method, appropriate metabolites were selected for pathway analysis to determine the potential metabolic pathways and mechanisms.

Tumor progression is a complex process involving proliferation, hypoxia, angiogenesis, apoptosis, metastasis, immunity, and increased tolerance to reactive oxygen species (Townson et al., 2003; Colin et al., 2014; Schito and Semenza, 2016; Karsch-Bluman et al., 2019; Messmer et al., 2019). These tumor-associated processes significantly affect primary metabolic pathways; Thus, it is primarily metabolic alterations that distinguish tumor cells from normally differentiated cells. In terms of lipid metabolism, tumor metabolites are characterized by an increase in lipid content, which happens to be necessary for the construction of cell membranes. Phospholipids are the main components of cell membranes and maintain the shape and fluidity of cells. Alterations in membrane phospholipids may be critical in influencing cancer phenotypes such as invasiveness and metastatic potential (Lavie et al., 1999).

Phospholipids are divided into two main groups, glycerophospholipids (GPs) and sphingophospholipids. Depending on the different substituents at the sn-3 position of the glycerol backbone, GPs fall into phosphatidylcholine (PC), phosphatidylethanolamine (PE), phosphatidyl glycerol (PG), phosphatidylserine (PS), phosphatidylinositol (PI), phosphatidic acid (PA), and cardiolipins. There is evidence that PC, PE and sphingomyelin (SM) are major components of eukaryotic cell membranes.

PE is a key phospholipid that helps maintain cell membrane fluidity. Lee et al. (2019) found that the concentrations of PE (36:1), PE (36:3), PE (38:6), and PE (18:0p/20:4) were increased in papillary thyroid cancer patients, but the changes of PE (38:3), PE (38:4), PE (40:6), and PE (18:0p/20:4) were in opposite directions in papillary thyroid cancer patients. In our study, PE (16:0/20:2), PE(O-18:0/18:3), PE(O-18:0/20:5), and PE (P-18:0/18:2) levels were down regulated in papillary thyroid cancer patients, however, PE-NMe (18:1/18:1) was in opposite directions in papillary thyroid cancer patients. PE is closely related to the regulation of calcium transport in cell signaling (Kester and Sokolove, 1990). In thyroid cancer cells, calcium transport is remodeled to provide help for cell proliferation and invasion (Gumbiner, 2005).

SM is an important component of biofilm composition. SM and its metabolites such as ceramide (Cer), sphingosine (Sph), and sphingosinephosphate (S1P) are an important class of biologically active signaling molecules involved in the regulation of many important signal transduction processes such as cell growth, differentiation, senescence and death are involved (Perry, 1999). Among them, Cer is the central molecule of SM metabolism, which together with Sph is a negative regulator of cell proliferation and can inhibit cell growth and promote apoptosis, while S1P stimulates cell growth and inhibits cell apoptosis. Together, they form a dynamic system of “Sphingolipid Rheostat” (Kohama et al., 1998; Hannun and Obeid, 2002). Previous studies found SM(d18:0/16:1) was significantly higher in thyroid papillary carcinoma than in normal thyroid tissue (Ishikawa et al., 2012). In our study, the levels of SM(d18:1/15:0), SM(d18:1/16:1), and SM(d16:1/24:1) were increased in patients with thyroid cancer patients, which seems to be inconsistent with the previous report. We believe that this may be related to the dynamic balance of sphingolipid variable blockers, and the deeper mechanism needs to be investigated further.

In cells, PC is mediated by phospholipase A2 (PLA2), a family of enzymes that hydrolyze glycerophospholipids to fatty acids and lysophosphatidylcholine. PLA2 is significantly more active in thyroid cancer cells than in normal thyroid tissue, and thus PC, along with its choline metabolites produced during metabolism, has an important role in tumor proliferation and survival (Cummings et al., 2000; Laye and Gill, 2003). Guo et al. (2015) found that PC (38:6) in plasma was significantly lower in malignant thyroid cancer than in healthy controls. Accordingly, our study also showed that PC(O-14:0/15:0) was down regulated in thyroid cancer patients. They are down-regulated probably due to higher rates of utilization as a result of increased demand for the membrane biosynthesis of tumor cells (Yang et al., 2017). It is consistent with some of the previous findings and is thought to be potentially relevant to the biological behavior of thyroid cancer.

Various glycosphingolipids were first hydrolyzed to glucosylceramide by glucoencephalosidase and glucosidase in lysosome and then converted to ceramide (Hannun and Obeid, 2002; Yuan et al., 2017). Ceramide is the central molecule of phospholipid metabolism, which mainly regulates the anti-proliferation effect. Such as inhibiting cell growth, inducing apoptosis, regulating senescence and autophagy. In the present study, the level of GlcCer (d14:1/24:1) was significantly increased in patients with thyroid cancer, which may be related to the fact that Ceramide can inhibit tumor growth by regulating the direct target of tumor growth and up-regulate the *de novo* synthesis of ceramide pathway Enzymes can reverse drug resistance in cancer cells (Wątek et al., 2019).

As an essential fatty acid, Alpha-Linolenic acid (ALA) mainly exists in body tissues in the form of complex lipids. The research results of Sauer et al. (2000). It is considered that the decrease of cell uptake of LA and its gene mutation enters the mitotic factor 13-hydroxyoctadecadienoic acid (13-HODE), thus inhibiting the growth of tumor. The study of Kato et al. (2002). found that the average tumor weight of nude mice inoculated with human colon cancer cells was significantly decreased in the high herring oil feed group and high alga oil feed group compared with the two control groups after 53 days of eating different diets, fully confirming that N-3 fatty acids can significantly inhibit tumor growth.

Linoleic acid, as an unsaturated fatty acid, has many functions. First, LA inhibits tumors by inducing the formation of lipid peroxidation products (Chen et al., 2001). Furthermore, LA can inhibit tumor formation through lipid metabolism (Sirui Mo, 2017). Finally, LA can induce the apoptosis of tumor cells (Yuan et al., 2009).

Glycosylated phosphatidylinositol (GPI) proteins are proteins that are anchored to the surface of eukaryotic cell membranes by a glycosylated phosphatidylinositol-anchored structure at the carboxyl terminus. GPI ethanolamine phosphate transferase participates in glycosylphosphatidylinositol biosynthesis. The relationship between GPI and tumor. First, GPI-anchored proteins are associated with tumor markers (Yinguang et al., 2009). GPI-anchored proteins are also involved in tumor cell signal transduction (Shumin et al., 2007). Furthermore, GPI-anchored proteins are associated with tumor metastasis (Philippova et al., 2005). Finally, GPI-anchored proteins are also involved in immune escape from tumors (Ge et al., 2016).

According to the current study, sphingolipids are the most recognized lipid markers. Sphingolipids play an important role in cell proliferation, migration, inflammatory response to anticancer drugs and other cancer-related functions as well as in preventing the occurrence and development of cancer (Canals et al., 2011). but no sphingolipid metabolism pathway was found in our pathway analysis, which may be due to the problem of the samples we selected. In addition, our sample size is relatively small, because we matched the basic clinical information, so the sample size is too small.

We didn’t find the sphingolipid pathway in our metabolic pathway, but phospholipids and glycolipids are complex lipids composed of simple lipids and non-lipid components (phosphoric acid, sugar, base, etc.). Phospholipids are lipids containing phosphoric acid. They can be divided into glycerol phospholipids and sphingosine phospholipids according to the different alcohols in the molecules. In addition, these two pathways can be interrelated through phosphoethanolamine and ceramide. In future experiments, we will expand the sample size and carefully screen the samples for further verification of the results. We would like to explore the role and association of sphingolipid metabolism in cancer.

This study culminated in the design of a predictive model that was constructed using altered lipid metabolites found previously in several patients with thyroid cancer, which will hopefully help in future work to diagnose thyroid cancer. Early diagnosis can enable PTC patients to receive more effective follow-up monitoring, especially high-risk patients to receive timely treatment to reduce the higher medical costs and physical injuries caused by delayed progression of the disease. Although ultrasound is the preferred method for the diagnosis of thyroid nodules, its application in the differential diagnosis of benign thyroid nodules and papillary thyroid carcinoma is controversial, and the diagnostic accuracy range is between 20 and 76% (Lin et al., 2015). The application of the gene expression abnormalities approach to cancer also has drawbacks. For example, The physiological reproducibility varied significantly among the various tumor heterogeneity features under investigation, only a few of them being identified as reproducible (Tixier et al., 2012). Since these lipid metabolites are common indicators, we believe that this diagnostic tool will be easily generalized and applied. However, it needs to be verified by expanding the sample size.

The limitations of our study include a relatively small sample size and a study group. Follicular, anaplastic, and poorly differentiated tumor samples were not included in our study because of their low incidence. In this study, we did not compare the changes in the lipid spectra of rai-refractory and rai-responsive. The samples selected in this study were patients with papillary thyroid carcinoma confirmed by pathology. There is no clear distinction between early-stage (I-II) and late-stage (III-IV) tumors. Another limitation of this study is the small data set. In the future, the sample size should be enlarged.

## Conclusion

The lipids in the serum of patients with PTC and in the healthy control groups were comprehensively analyzed using UPLC-QTOF/MS. Thirteen lipid species are proposed as potential biomarkers for the diagnosis of PTC. These species showed significant differences between the PTC and healthy control group. The identified biomarker or panels showed excellent diagnostic accuracies for distinguishing among PTC patients, and normal individuals. The predictive model showed good diagnostic performance and it could be gradually incorporated as a support method for the diagnosis of PTC.

## Data Availability Statement

The original contributions presented in the study are included in the article/supplementary material, further inquiries can be directed to the corresponding author/s.

## Ethics Statement

The study was performed according to the standards of the Institutional Ethical Committee and the Helsinki Declaration of 1975, as revised in 1983, and was approved by the Institutional Review Board of the Tsinghua University. The patients/participants provided their written informed consent to participate in this study.

## Author Contributions

NJ, ZZ, and XC were involved in the study concept and design. NJ, ZZ, GZ, LP, CY, GY, and LZ provided the tools and patient specimens. XC and YW performed the experiments. XC, JH, and TX analyzed and interpreted the results and edited the manuscript. JH and TX organized the results and drafted the manuscript. NJ and XC approved the final version. All authors participated in the critical revision of the manuscript for important intellectual content.

## Conflict of Interest

XC and JH were employed by the BaoFeng Key Laboratory of Genetics and Metabolism. TX was employed by the Zhongguancun Biological and Medical Big Data Center. The remaining authors declare that the research was conducted in the absence of any commercial or financial relationships that could be construed as a potential conflict of interest.
